# Preoperative radiotherapy improves survival in rectal signet-ring cell carcinoma-a population-based study

**DOI:** 10.1186/s13014-017-0874-0

**Published:** 2017-08-23

**Authors:** San-Gang Wu, Wen-Wen Zhang, Jia-Yuan Sun, Zhen-Yu He, Guo-Qiang Su, Feng-Yan Li

**Affiliations:** 1 Department of Radiation Oncology, Xiamen Cancer Hospital, The First Affiliated Hospital of Xiamen University, Xiamen, 361003 People’s Republic of China; 2 Department of Radiation Oncology, Sun Yat-sen University Cancer Center, State Key Laboratory of Oncology in South China, Collaborative Innovation Center of Cancer Medicine, Guangzhou, 510060 People’s Republic of China; 3 Department of Gastrointestinal Surgery, Xiamen Cancer Hospital, the First Affiliated Hospital of Xiamen University, Xiamen, 361003 China

**Keywords:** Rectal cancer, Signet-ring cell carcinoma, Radiotherapy, Preoperative, Survival

## Abstract

**Background:**

To investigate the clinical value of preoperative and postoperative radiotherapy (RT) in patients with rectal signet-ring cell carcinoma (SRCC).

**Methods:**

Using the Surveillance, Epidemiology, and End Results program patients with stage II–III rectal SRCC were retrospectively included between 1988 and 2012. Univariate and multivariate Cox regression analyses were performed to analyze the effect of preoperative and postoperative RT on cause-specific survival (CSS).

**Results:**

A total of 292 patients were included: 138 patients received preoperative RT, 101 patients received postoperative RT, and 53 patients underwent surgery alone. Overall, 5– and 10-year CSS was 43.8 and 37.6%, respectively. Preliminary survival analysis demonstrated that preoperative RT improved CSS versus surgery alone, especially in patients with stage III disease. Multivariate analysis demonstrated that preoperative RT was independent predictors for CSS in stage III rectal SRCC. CSS in preoperative and postoperative RT groups was comparable.

**Conclusions:**

Preoperative RT significantly improved survival outcomes in patients with stage III rectal SRCC.

## Background

Colorectal cancer (CRC) is one of the commonest cancers worldwide, and a variety of histological subtypes have been reported [1, 2]. Signet-ring cell carcinoma (SRCC) is a rare variant of adenocarcinoma, found in approximately 1% of all CRC patients and presents with abundant intracellular mucin in more than 50% of tumor cells, rendering their signet-ring appearance [3–6]. SRCC histology is considered an adverse risk factor in esophageal, stomach, and breast cancer [7–9]. Due to its rarity SRCC has been investigated in a limited number of studies with small samples of patients. SRCC is associated with a poor prognosis and higher risk of death compared with colorectal adenocarcinoma without signet-cell histology [3, 5, 6, 10–12].

The optimal treatment strategy of colorectal SRCC features a multidisciplinary approach taking into consideration the natural history of these tumors and tumor-related prognostic factors. International clinical practice guidelines do not recommend specific treatment for SRCC histology [13, 14]. However, existing evidence shows that colorectal SRCC responds poorly to cytotoxic therapies and has a low rate of curative resection and poor survival; hence new treatment approaches are needed [10]. Preoperative or postoperative concurrent chemoradiation therapy (CCRT) is standard treatment for locally advanced rectal cancer (stage II/III). SRCC tends to present with advanced tumor stage and nodal involvement [15, 16]. Therefore radiotherapy (RT) may be of potential clinical value in this entity. However, in patients with cervical cancer and esophageal adenocarcinoma, SRCC histology seems associated with resistance to RT/CCRT [17, 18]. In this study we examined data from a population-based cancer registry (Surveillance, Epidemiology, and End Results; SEER) to assess the effect of preoperative and postoperative RT in patients with rectal SRCC.

## Materials and methods

### Patients

This study used the SEER database to investigate the clinical value of preoperative and postoperative RT against rectal SRCC. The SEER dataset is maintained by the US National Cancer Institute and consists of 18 population-based cancer registries that include information on cancer incidence and mortality in the USA [19]. From the database patients with the following included criteria were enrolled: 1) diagnosis of stage II–III rectal SRCC (site code, C20.9; histology code, 8490/3) between 1988 and 2012; 2) surgical resection of primary tumor either alone or with RT prior to or after surgery. Patients with an unknown number of removed lymph nodes (RLNs) and positive lymph nodes (PLNs) were excluded. This study was approved by the ethics committees of the First Affiliated Hospital of Xiamen University.

### Demographic and clinicopathological variables

Patients’ demographic and clinicopathological variables were retrieved from the SEER database, including sex, age at diagnosis, race/ethnicity, histological grade, tumor size, tumor stage, number of RLNs and PLNs, and local treatment strategy. The primary endpoint was cause-specific survival (CSS).

### Statistical analysis

A comparison of the categorical variables among the three treatment groups was performed using Pearson’s χ2 test, except for continuous variables, for which an analysis of variance (ANOVA) was used. Survival curves were calculated by Kaplan–Meier method and compared by log-rank test. The Cox proportional hazard regression model was used to identify risk factors that could independently influence CSS in rectal SRCC. All statistical analyses were performed using IBM SPSS software (version 21.0; IBM Corporation, Armonk, NY, USA); a *p*-value < 0.05 indicated statistical significance.

## Results

**Fig. 1:**
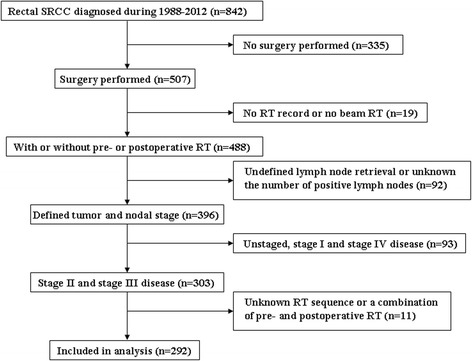
The flow chart of the study

A total of 292 SRCC patients were included. Figure 1 depicts the flow chart of the study. Their median age at diagnosis was 58 (range, 17–90) years. Most of the patients were white and diagnosed with poorly differentiated/undifferentiated tumors. Patient demographics and clinicopathological characteristics are summarized in Table 1. A total of 51 patients (17.5%) had stage II disease and 241 (82.5%) stage III disease. In all, 138 patients (47.3%) received preoperative RT and 101 (34.6%) postoperative adjuvant RT; 53 patients (18.2%) received surgery alone. Patients receiving preoperative and postoperative RT were somewhat younger than those who received surgery alone (median age, 54 [range, 17–88] vs. 58 [range, 21–88] vs. 73 [range, 28–90] years; *p* < 0.001). A higher number of PLNs was also found in patients who received surgery alone compared with patients who received preoperative RT (median PLNs, 6 [range, 0–51] vs. 3 [range, 0–29]; *p* = 0.019). There was no significant difference of sex, tumor stage, tumor grade, tumor size, and RLN count among the three treatment groups.

**Table 1:** Patient characteristics

Characteristic	n	Radiotherapy			*p*
		No (%)	Preoperative (%)	Postoperative (%)	
Sex					
Male	198	35 (66.0)	89 (64.5)	74 (73.3)	0.341
Female	94	18 (34.0)	49 (35.5)	27 (26.7)	
Age					
Mean ± SD (years)	57 ± 16.8	67 ± 17.2	54 ± 16.3	55 ± 15.2	<0.001
Race					
White	224	40 (75.5)	99 (71.7)	85 (84.2)	0.095
Black	23	5 (9.4)	10 (7.2)	8 (7.9)	
Other and unknown	45	8 (15.1)	29 (21.0)	8 (7.9)	
Tumor grade					
G1–2	20	3 (5.7)	7 (5.1)	10 (9.9)	0.118
G3–4	237	47 (88.7)	108 (78.3)	82 (81.2)	
Unknown	35	3 (5.7)	23 (16.7)	9 (8.9)	
Tumor size (mm)					
Mean ± SD	53.5 ± 25.1	55.4 ± 26.6	53.3 ± 25.6	52.8 ± 23.9	0.835
Number of RLNs (n)					
Mean ± SD	14.8 ± 11.6	13.3 ± 10.0	13.9 ± 10.1	16.9 ± 13.9	0.080
Number of PLNs (n)					
Mean ± SD	6.7 ± 7.2	8.3 ± 9.2	5.2 ± 5.8	7.9 ± 7.2	0.003
Tumor stage					
II	51	11 (20.8)	29 (21.0)	11 (10.9)	0.099
III	241	42 (79.2)	109 (79.0)	90 (89.1)	

*G1* well differentiated, *G2* moderately differentiated, *G3* poorly differentiated, *G4* undifferentiated, *PLNs* positive lymph nodes, *RLNs* removes lymph nodes, *SD* standard deviation

**Fig. 2:**
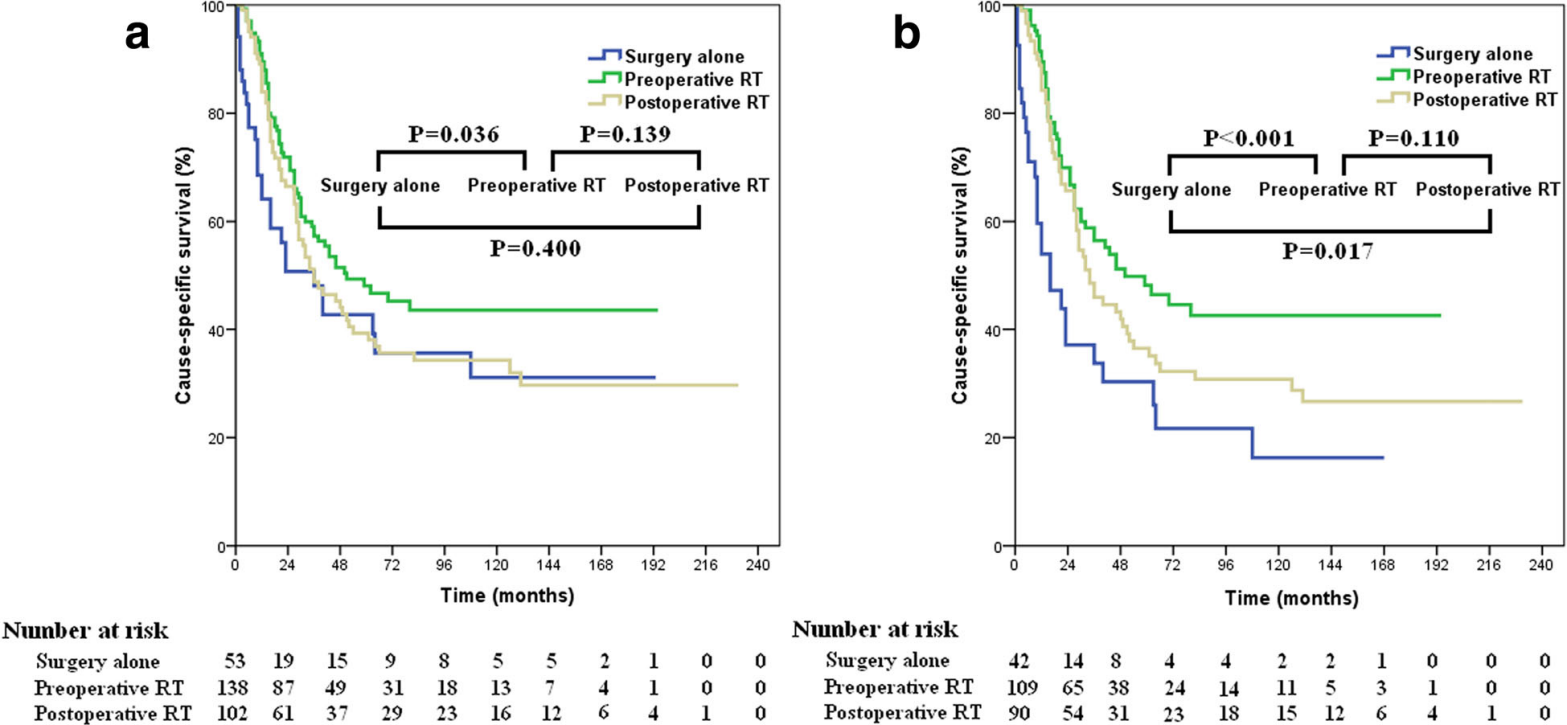
Impact of local treatment strategy on cause-specific survival in entire cohort (**a**) and stage III disease (**b**)

The median follow-up was 29 (range, 0–231) months. Five-year and 10-year CSS was 43.8 and 37.6%, respectively. The 5-year and 10-year overall survival (OS) was 39.0 and 30.1%, respectively. The 5-year CSS of patients in preoperative RT, postoperative RT, and surgery alone groups was 48.1, 39.3, and 42.7%, respectively (*p* = 0.086) (Fig. 2a). Subgroup analysis indicated that preoperative and postoperative RT improved CSS in stage III (48.2% vs. 36.5% vs. 30.4%; *p* = 0.001) (Fig. 2b), but not in stage II rectal SRCC (*p* = 0.152). CSS in preoperative and postoperative RT groups was not significantly different in the entire cohort (*p* = 0.139), as well as those stratified by stage II (*p* = 0.571) and stage III disease (*p* = 0.110).

RT sequence, age at diagnosis, race, tumor size, tumor stage, and number of PLNs were significant prognostic factors for CSS in univariate analysis (Table 2). Demographic and clinicopathological factors that were significantly associated with poor CSS in univariate analysis were included in the multivariate Cox analysis; age, tumor size, and tumor stage were thereby revealed as independent prognostic factors of survival. Preoperative and postoperative RT was not an independent predictor for CSS (Table 2). However, in the subgroup analysis of patients with stage III rectal SRCC, the results of multivariate Cox analysis showed that treatment type was an independent prognostic factor for survival outcomes; compared with surgery alone group, stage III patients receiving preoperative RT (hazard ratio [HR] 0.467, 95% confidence interval [CI] 0.274–0.794, *p* = 0.005] had better CSS compared with patients who received surgery alone, while postoperative RT (HR 0.615, 95% CI 0.374–1.011, *p* = 0.055] had similar CSS compared with patients who received surgery alone (Table 3).

**Table 2:** Univariate and multivariate analyses on prognostic factors of cause-specific survival in all patients (*n* = 292)

Characteristic	Univariate analysis		Multivariate analysis	
	HR (95% CI)	*p*	HR (95% CI)	*p*
Sex				
Male	1		—	
Female	1.299 (0.940–1.794)	0.113	—	—
Age	1.012 (1.002–1.022)	0.019	1.017 (1.005–1.030)	0.007
Race				
White	1		1	
Black	1.698 (1.004–2.871)	0.048	1.802 (0.974–3.334)	0.061
Other and unknown	1.247 (0.809–1.924)	0.317	1.524 (0.930–2.495)	0.094
Tumor grade				
G1–2	1		—	
G3–4	0.850 (0.470–1.537)	0.590	—	—
Tumor size (mm)	1.016 (1.009–1.023)	< 0.001	1.017 (1.009–1.025)	< 0.001
Number of RLNs	0.997 (0.984–1.010)	0.651	—	—
Number of PLNs	1.047 (1.026–1.068)	< 0.001	1.021 (0.995–1.048)	0.120
Tumor stage				
II	1		1	
III	1.922 (1.189–3.108)	0.008	2.289 (1.170–4.478)	0.016
Treatment				
Surgery alone	1		1	
Preoperative RT	0.627 (0.403–0.978)	0.039	0.656 (0.391–1.100)	0.110
Postoperative RT	0.812 (0.520–1.268)	0.359	0.788 (0.470–1.323)	0.368

*CI* confidence interval, *G1* well differentiated, *G2* moderately differentiated, *G3* poorly differentiated, *G4* undifferentiated, *HR* hazard ratio, *PLNs* positive lymph nodes, *RLNs* removes lymph nodes, *RT* radiotherapy

**Table 3:** Univariate and multivariate analyses on prognostic factors of cause-specific survival in patients with stage III disease (*n* = 239)

Characteristic	Univariate analysis		Multivariate analysis	
	HR (95% CI)	*p*	HR (95% CI)	*p*
Sex				
Male	1		—	
Female	1.257 (0.890–1.776)	0.195	—	—
Age	1.013 (1.003–1.024)	0.012	1.013 (1.000–1.025)	0.045
Race				
White	1		—	
Black	1.463 (0.804–2.665)	0.213	—	—
Other and unknown	1.037 (0.649–1.659)	0.879	—	—
Tumor grade				
G1–2	1		—	
G3–4	0.616 (0.332–1.143)	0.125	—	—
Tumor size (mm)	1.017 (1.010–1.025)	<0.001	1.019 (1.012–1.027)	<0.001
Number of RLNs	0.996 (0.983–1.010)	0.608	—	—
Number of PLNs	1.041 (1.018–1.064)	0.001	1.016 (0.990–1.043)	0.222
Treatment				
Surgery alone	1		1	
Preoperative RT	0.418 (0.262–0.667)	<0.001	0.467 (0.274–0.794)	0.005
Postoperative RT	0.565 (0.358–0.892)	0.014	0.615 (0.374–1.011)	0.055

*CI* confidence interval, *G1* well differentiated, *G2* moderately differentiated, *G3* poorly differentiated, *G4* undifferentiated, *HR* hazard ratio, *PLNs* positive lymph nodes, *RLNs* removes lymph nodes, *RT* radiotherapy

## Discussion

In this study, we analyzed the SEER database to investigate the clinical value of preoperative and postoperative RT in patients with stage II–III rectal SRCC and found that, at least in stage III patients, preoperative RT was associated with better survival outcomes than surgery alone. However, we did not find any survival benefit of preoperative and postoperative RT in stage II rectal SRCC.

SRCC of the rectum is a rare malignant tumor and highly aggressive in CRC. A study using the National Cancer Data Base showed that rectal SRCC histology was independently correlated with a greater risk of death (*n* = 448) [3]. However, 5-year survival rate differed greatly according to tumor stage at diagnosis [3]. Studies from The Netherlands and Asian countries showed that 5-year survival in patients with SRCC of the colon and rectum stage II and III was 27.4–100 and 14.5–32.5%, respectively [10, 11, 15, 20]. In these reports differences in the number of patients may have influenced the large disparities of survival in stage II patients. In our population-based study from the SEER database, most patients were stage III and 5-year CSS in patients with stage II and III disease was 58.5 and 40.5%, respectively. The 5-year OS was 53.5 and 35.8%, respectively. SRCC histology is associated with high histological grade and advanced tumor and nodal stage compared with mucinous carcinoma and well/moderately/poorly differentiated adenocarcinoma [15, 16]. It was also found that the rates of angioinvasion and lymphatic invasion are higher in colorectal SRCC subtype [20]. Therefore the intrinsic tumor biology of SRCC may contribute to its aggressive clinical behavior and dismal prognosis.

Preoperative and postoperative CCRT is standard treatment for locally advanced rectal cancer. In squamous cell carcinoma of the uterine cervix, patients with “immature glandular features” including signet ring cells are known as an independent predictor of radiation resistance [17]. In patients with esophageal adenocarcinoma SRCC histology is associated with reduced complete response rates and poor survival in patients undergoing preoperative CCRT [18]. These findings suggest that SRCC histology may confer resistance to radiotherapy/chemotherapy. However, no large studies have assessed the efficacy of RT alone or in combination with chemotherapy in SRCC tumors. A study showed good pathological response rates in patients with rectal SRCC after preoperative CCRT, and SRCC histology was found independently predictive of treatment response in multivariate analysis [21]. However, in that study there were only 5 patients with SRCC histology [21], and the clinical value of RT in rectal SRCC remains unknown.

For a rare disease such as rectal SRCC, it is difficult to include sufficient numbers of patients treated in a single center. Therefore we carried out a population-based study using the SEER database to determine the impact of preoperative and postoperative RT in patients with rectal SRCC. We found that treatment using these interventions conferred meaningful survival benefit only in patients with stage III and not stage II disease, and preoperative RT was an independent predictor for CSS.

To the best of our knowledge, the present study is the first and largest to assess the effects of preoperative and postoperative RT for rectal SRCC. Randomized controlled trials have found that preoperative CCRT improved local control and was associated with reduced toxicity but did not improve OS in comparison with patients who received postoperative CCRT [22–24]. However, preoperative CCRT was associated with significantly improved histopathologic downstaging, resectability rate, and sphincter preservation in low-lying rectal cancer. Nowadays, preoperative CCRT has become widely accepted worldwide. In the present study, preoperative RT improved CSS in stage III rectal SRCC, and CSS in preoperative and postoperative RT groups was comparable. Although SEER database lacks the assessment of treatment response after preoperative RT. However, a study by Jayanand et al. indicated that rectal SRCC was an independent predictor of pathological complete response (pCR) (*p* = 0.001), and pCR was associated with better local control and OS [21]. Bertland et al. also found that preoperative RT was associated with better tumor response in locally advanced or recurrent rectal SRCC [25]. Therefore, preoperative RT may confer survival advantage in rectal SRCC.

Although our study was a population-based SEER analysis, it should be acknowledged that there are several limitations in our study. First, its retrospective design incurs inherent bias. Second, the SEER database lacks important information including centralized pathologic review, quality of surgery, details of RT and chemotherapy, uniformity in treatment, pathological response to RT, patterns of local and distant recurrence, and treatment complications. In addition, patients with preoperative and postoperative RT were younger and may have had better health and less comorbidity, which could also confound the results.

## Conclusion

In conclusion, preoperative RT significantly improved survival outcomes in patients with stage III rectal SRCC. We believe our study may be of note for RT centers that treat rectal SRCC. Further prospective studies are needed to confirm our results.

## Abbreviations

ANOVA: Analysis of variance
CCRT: Concurrent chemoradiation therapy
CI: Confidence interval
CRC: Colorectal cancer
CSS: Cause-specific survival
HI: Hazard ratio
pCR: Pathological complete response
PLNs: Positive lymph nodes
RLNs: Removed lymph nodes
RT: Radiotherapy
SEER: Surveillance, Epidemiology, and End Results
SRCC: Signet-ring cell carcinoma
